# Clinical and laboratory characteristics of adolescents with platelet function disorders and heavy menstrual bleeding

**DOI:** 10.1186/2162-3619-2-3

**Published:** 2013-01-24

**Authors:** Lawrence S Amesse, Teresa Pfaff-Amesse, William T Gunning, Nancy Duffy, James A French

**Affiliations:** 1 Division of Reproductive Endocrinology and Infertility, Department of OB-GYN, Section of Pediatric-Adolescent Gynecology, Wright State University Boonshoft School of Medicine, Dayton, OH, USA; 2 Division of Pediatric Hematology & Oncology, Department of Pediatrics, Wright State University Boonshoft School of Medicine, Dayton, OH, USA; 3 Department of Pathology, University of Toledo College of Medicine, Dayton, OH, USA

**Keywords:** Platelet function disorders, Adolescents, Heavy menstrual bleeding, Menorrhagia, δ-Storage Pool Deficiency (δ-SPD)

## Abstract

**Background:**

Platelet function disorders (PFDs) have emerged as an important etiology of heavy menstrual bleeding (HMB) in adolescents. However, neither clinical nor laboratory data have been methodically analyzed in this population subset. The objective of this study was to evaluate these parameters in order to distinguish characteristics of the disorder that in turn will lead to earlier diagnosis and therapy initiation.

**Methods:**

Retrospective review of medical records from postmenarcheal adolescents with documented PFDs referred to a hemophilia treatment center and university faculty practices for bleeding diatheses with their clinical and laboratory data evaluated.

**Results:**

Of 63 teens with documented PFDs, HMB was the most common clinical manifestation of PFD (43; 68.3%). Of these, 37 (86%) were diagnosed with PFD either at or after menarche with the diagnosis based on HMB symptoms alone. Only 6 (14%) were diagnosed with a PFD prior to menarche, based on associated bleeding, i.e., epistaxis, ecchymosis, and all developed HMB after menstruation onset. Interestingly, 20 girls were diagnosed with a PFD prior to menarche and of these, only 6 (30%) went on to develop HMB after pubertal transition, while the majority (14; 70%) did not. The average age-at-PFD diagnosis was 14.5yrs, significantly differing from the 10.9yrs average age-at-PFD diagnosis in their counterparts that, after menarche, did not develop HMB (*P*<.01) Blood type O occurred significantly more frequently (76%) than national norms (*P* <.037). Incidence of δ-Storage Pool deficiency (δ-SPD) was significantly higher (74%) than their non-HMB cohorts (45%) (*P* <.007). Coagulation and von Willebrand factor studies were all normal. Abnormal closure times and aggregation studies were observed in 42% and 60%, respectively, of tested girls. In 25.6% for whom standard platelet studies were normal, electron microscopy detected reduced platelet δ-granules numbers (δ-SPD).

**Conclusions:**

Adolescents with PFDs and HMB appear to be clinically distinct from their non-HMB counterparts. This group of girls is characterized by HMB the major bleeding symptom, significantly high incidences of blood group O and the δ-SPD with a PFD diagnosed well after menarche. High false negative standard platelet function study results indicate additional diagnostic strategies, particularly for δ-SPD, should be considered.

## Background

Abnormal uterine bleeding is a common problem in the adolescent population, where it affects up to 37% of teenage girls [1,2]. The bleeding etiology in this age group is often attributed to anovulatory menstrual cycles subsequent to immaturity of the hypothalamic-pituitary-ovarian axis [3,4]. However, in nearly one half of all cases a definitive etiology remains elusive [5-13]. Hemostatic disorders such as von Willebrand disease (VWD) and single coagulation factor deficiencies have been implicated as important etiologies for many years.

Platelet function disorders (PFDs)--a heterogeneous group of inherited, qualitative platelet defects--have recently emerged as a frequent, equally important cause of abnormal uterine bleeding in adolescents. In this population subset, PFDs often manifest as heavy menstrual bleeding (HMB), formerly termed menorrhagia, defined as menstrual blood loss >80mL per menses [1,4-7,9-17]. However, platelet dysfunction-associated HMB has not been methodically analyzed in adolescents, and there is limited published objective data on the disorder. For the last decade, a total of only 2 communications have examined in detail the clinical and laboratory features of platelet dysfunction-associated HMB in teenagers, and for both studies, data was obtained from a total of 10 adolescents combined [5,7]. Indeed, most reports on HMB intermixed demographic data from adolescents with PFDs with either similarly affected adults or adolescents having disparate hematologic disorders, without exclusively evaluating adolescents with PFDs and HMB [4,6,8-16,18]. Other reports and case series were predominately composed of patients diagnosed with syndromic platelet disorders, e.g., Bernard Soulier syndrome, Glanzmann’s thrombasthenia, or idiopathic thrombocytopenic purpura [4,19,20].

Platelets are integral components of primary hemostasis. Upon vascular injury, von Willebrand factor (VWF) mediates platelet adherence to the exposed subendothelial matrix. Platelets are then activated and secrete the contents of their alpha (α) and dense (δ) granules and other factors, i.e., thromboxane A2, platelet activating factor. The secreted factors in turn interact with specific platelet receptors to mediate activation and recruitment of additional platelets that bind to the adhered platelets, forming aggregates that lead to the formation of a hemostatic plug.

Inherited PFDs can be classified according to their functional defects, although clear distinctions are not always evident. Defects can involve platelet secretion (e.g. α-, δ-granule storage pool deficiencies); signal transduction pathways (e.g. arachidonic acid, thromboxane A2); signal transduction receptors (e.g., thromboxane A2, collagen); and platelet adhesion receptors (e.g., glycoprotein Ia/IIa). Of these, the dense (δ) granule storage pool deficiency (SPD) is the most common.

A wide range of specialists evaluate young girls presenting with HMB. The initial evaluation includes a careful history with directed attention to personal and familial bleeding tendencies. Pertinent examination findings include petechiae and ecchymosis. These teenagers should undergo platelet testing concurrent with VWF assays. Initial laboratory studies include complete blood count (CBC) with platelet counts and morphology, prothrombin time (PT), activated partial thromboplastin time (aPTT) and fibrinogen or thrombin and VWF tests for VWD diagnosis [ristocetin cofactor (VWF:RCo), VWF antigen (VWF:Ag), VWF:RCo / VWF:Ag ratios and factor VIII activity (F VIII:C)]. However, no single laboratory test can diagnose all platelet disorders and consultation with a hematologist is often indicated. Rapid, accurate diagnosis of PFDs in young girls clinically manifesting HMB is crucial to initiation of appropriate therapy that in turn prevents severe bleeding, emergent hospitalizations, blood transfusions and bleeding at surgery. Early detection also mitigates the negative impact HMB has on an adolescent’s education as well as reduced lifestyle and quality of life parameters [9].

In this communication we focus exclusively on a subset of adolescents with documented PFDs and a physician diagnosis of HMB. Here we report on various clinical and laboratory findings that offer novel insights into the early natural history of the disorder. These data provide a foundation for understanding platelet dysfunction-associated HMB in this population subset that will aid in it’s earlier recognition, diagnosis and therapeutic initiation.

## Results

Seventy-one young females with objectively documented PFDs were initially identified; 8 were disqualified for reasons of menarche not achieved (6) and failure to return for follow-up visits (2). The study population was composed of a cohort of 63 postmenarcheal adolescents with bleeding symptoms and documented PFDs. Their clinical and laboratory profiles are summarized in Table 1.

**Table 1:** Clinical and laboratory characteristics at presentation for postmenarcheal adolescents with documented platelet function disorders

**Clinical & Laboratory Characteristics**	**Heavy Menstrual Bleeding (n=43)**			**Non-Heavy Menstrual Bleeding (n=20)**			***P***
Age (y) at PFD diagnosis (mean; range)	14.5 ± 3.5; 3 - 20			10.9 ± 3.5; 5 - 17			<.01
Prior to menarche	6 (14)			14 (70)			
At menarche	10 (23)			0			
After menarche	27 (63)			6 (30)			
Age (y) at menarche (mean; range)	11.8 ± 1.0; 9 - 15			12.1 ± 1.5; 9 - 16			.36
Interval (y): PFD-dx-to menarche (mean)	2.54 ± 3.09			1.48 ± 3.11			<.01
	*after* menarche			*prior* to menarche			
Age (y) at HMB onset (mean; range)	13.5 ± 1.9; 9 - 18			0			
At menarche	10 (23)			0			
After menarche	33 (77)			0			
Interval (y): from menarche-to-HMB onset (mean; range)	1.79 ± 1.4; 0–5.0			0			
PBAC score ≥ 100	43 (100)			0			
Ethnicity							
Caucasian	35 (81)			16 (80)			1.0
African-American	4 ( 9)			1 (5)			
Asian	1 ( 2)			0			
Hispanic	1 ( 2)			1 (5)			
Multiethnic	2 ( 5)			2 (10)			
Family History of Bleeding Tendencies							
Positive	36 (83.7)			17 (85)			.41
Negative	7 (16.3)			3 (15)			
	ABO	Rh(D)		ABO	Rh(D)		
Blood Group Types		+	**−**		**+**	**−**	
Group O	33 (76.0)	27	6	14 (70.0)	13	1	.037^†^
Group A	9 (21.0)	9	0	6 (30.0)	6	0	
Group B	1 ( 3.0)	1	0	0	0	0	
Hematocrit (%) (mean)*	37.3 ± 4.8			38.0 ± 3.9			.7
MCV (fL) (mean)^*^	85.4 ± 5.1			86.0 ± 5.9			.75
Platelet count (μ/L) (mean)	284.0 ± 62.3			285.0 ± 71.0			.99
PT (s) (mean)	12.2 ± 0.81			12.5 ± 0.94			.35
aPTT (s) (mean)	31.8 ± 6.3			31.4 ± 4.0			.82
			ABO-Adjusted	VWF Levels			
von Willebrand Factor Assays^‡^	O		A,B	O		A,B	
VWF:RCo (%) (mean)	69.7±18.7		91.9±16.2	84.5±21.2		101.2±29.6	.13
VWF:Ag (%) (mean)	88.0±10.3		100.7±13.6	88.3±10.1		104.0±24.6	
FVIII:C (%) (mean)	119.0 ± 66.7			104.0 ± 27.9			
VWF:RCo/VWF:Ag ratio (mean)	0.912 ± 0.235			0.960 ± 0.274			

aPTT=activated partial thromboplastin time; dx=diagnosis; FVIII:C=Factor VIII activity; MCV=mean corpuscular volume; PT= prothrombin time;VWF=von Willebrand factor; VWF:RCo=ristocetin cofactor activity; VWF:AG=VWF antigen; PBAC=pictorial bleeding assessment chart; U.S.=United States.

Data for both groups are presented as means ± standard deviations or n (%).

^*^Reference intervals (females) at corresponding ages ≤13yrs or >14yrs:Hematocrit, 35-45% or 37-47%; MCV, 78-95fL or 78-100fL; platelet count, 140,000-450,000μ/L.

^†^*P*-value applies only to ABO blood group distribution differences between HMB-group & a comparable U.S. population.^21,22^^‡^von Willebrand Factor Assay reference intervals adjusted for blood groups O & A,B: A,B:VWF:RCo=37.5-117.1 & 48 206.1;VWF:Ag=48.1-146 & 50.7-193.7, respectively.

### Clinical features

Heavy menstrual bleeding was the most common clinical manifestation of a PFD, 68%, (n=43) (HMB group). Thirty-two percent (n=20) had neither documentation nor a physician diagnosis of HMB after pubertal transition (non-HMB group). Of the 43, 86% were diagnosed with a PFD after menarche based on heavy menstrual bleeding symptoms, while only 14% were diagnosed with a PFD prior to achieving menarche. Of the 14%, platelet function studies were initiated based on associated bleeding symptoms, including epistaxis, easy bruisability, surgery-associated bleeding, etc. A total of 20 patients were diagnosed with a PFD prior to achieving menarche with only 6 (30%) manifesting HMB after the menarche, while the majority (13; 70%) did not develop HMB after menarche (median follow-up, 42mo). The average age-at-PFD diagnosis in patients affected with HMB was 14.5 years, with later age at diagnosis based on HMB the sole bleeding symptom in 86%, significantly differing from the average age-at-PFD diagnosis, 10.9 years, observed in PFD patients that did not develop HMB after menarche (*P*<.01).

Later age-at-PFD diagnosis is also observed when the menarche is considered a point of reference. The interval from age-at-PFD diagnosis to age-at-menarche in adolescents affected with HMB was on average 2.5 years after menarche, significantly at variance with the (mean) 1.5 years prior to menarche observed in patients that did not manifest HMB after pubertal transition. (*P<*.01). There were no significant differences between the 2 groups in (mean) age at menarche (*P*=.36).

Onset of HMB occurred at (mean) age 13.5 years with the interval between menarche-to-HMB onset (mean) 1.8 years, indicating that for a large majority, 33 (77%), HMB occurred after experiencing several menstrual cycles.

Caucasian (80-81%) was the most common ethnicity. Additional ethnicities in descending order of frequency included African American, “Multiethnic,” Hispanic and Asian. Over 80% of participants gave family histories positive for bleeding tendencies. No significant differences between the 2 groups were identified with respect to these parameters (*P*>.05).

### Laboratory features

Type O was the most common blood group and there were no significant differences identified between intergroup ABO-Rh(D) phenotypic frequencies (*P*>.05). However, teenagers affected with PFDs had ABO blood types that were at a significant variance with a comparable Caucasian U.S. population. The 70-76% type O frequency observed in our PFD teens occurred more frequently than the 44-45% reported for an analogous national population, while types A and B occurred less frequently. Type A was found in 20.9%-30% of our PFD adolescents compared with 40-42% reported for national Caucasian norms; type B, detected in only 0–2.3% versus 11% nationally (*P*=.037) [21,22]. The AB blood group, noted in 4% of the United States population, was not expressed by any PFD patients.

Adolescents with PFDs that manifested HMB were not anemic; the (mean) hematocrits, hemoglobin levels (not shown) and MCVs, all fell within normal ranges for their respective ages, ≤ 13yrs or >14yrs, and gender. Platelet counts and morphologic examination**,** coagulation studies and VWF studies for VWD diagnosis all fell within normal ranges for their respective ABO blood groups with no significant differences identified between the two groups for these parameters (*P*>.05). Abnormal VWF tests were recorded in only 1 adolescent (HMB group) diagnosed with combined PFD-VWD, type I. Factor II (1), V (1), VII (1), IX (1), XI (9) and XII (8) levels performed in selected patients all fell within normal ranges (not shown).

### Bleeding profile

The nature and number of bleeding symptoms at presentation for all postmenarcheal adolescents with documented PFDS are shown in Table 2. Heavy menstrual bleeding was the only presenting symptom in 37 (86%) of the HMB group. The majority 28 (65%) experienced additional bleeding symptoms, with 1 patient reporting 5 different symptoms. Additional bleeding included epistaxis (18; 64%), easy bruisability (16; 57%), surgery-associated bleeding (8; 29%), and gingival and/or mucosa bleeding (2; 4%). Only 6 (14%) presented with non-HMB bleeding symptoms and all did such prior to menarche. Non-HMB adolescents with PFDs shared a similar bleeding profile. No significant differences were identified between both groups with respect to the nature or the number of bleeding symptoms (*P*>.05).

**Table 2:** Bleeding profile of adolescents with platelet function disorders

	**Heavy Menstrual Bleeding (n=43)**	**Non-Heavy Menstrual Bleeding (n=20)**
Bleeding symptoms at presentation		
Heavy menstrual bleeding only	37 (86)	0
Other PFD-associated bleeding	6 (14)	20 (100)
Number of bleeding symptoms per patient		
one	15 (35)	11 (55)
two	23 (53)	5 (25)
three or more	5 (12)	4 (20)
Other PFD-associated bleeding symptoms:		
Epistaxis	18 (64)	14 (70)
Easy bruisability	16 (57)	10 (50)
Post operative bleeding associated with:	8 (29)	6 (30)
*Tonsillectomy; adenoidectomy*	4	2
*Dental surgery and/or extraction*	2	3
*Gynecological procedure/surgery*	1	0
*Not specified*	1	1
Gingival and/or oral mucosa	2 (4)	1 (5)
Hematuria	0	1 (5)

PFD=platelet function disorder *P*-values exceeded 0.05 for all differences between the two groups.

Data for both groups are presented as n (%).

### Platelet function studies

Abnormal testing results from platelet studies—prolonged closure times using the platelet function analyzer (PFA) system and impaired platelet aggregation by light transmission aggregometry (LTA)—and electron microscope (EM) detection of reduced platelet δ-granule numbers are summarized in Table 3 for both groups [7,11-13,23-26]. The majority of teens, 26 (60.5%), affected with HMB had one abnormal platelet study, while 17 (39.5%) had 2 abnormal studies. Similar results were observed in non-HMB adolescents: 14 (70%) and 6 (30%), 1 and 2 abnormal platelet studies, respectively. No patient had 3 abnormal platelet studies. The reciprocal observation indicated that 11 (25.6%) and 2 (10%) of HMB and non-HMB patients, respectively, had normal platelet function studies and for these patients, EM detection of diminished storage pool granule numbers was the only study that identified a platelet disorder. EM also identified similar findings in 14.3% of teens with HMB that had single agonist defects on platelet aggregation studies.

**Table 3:** Abnormal platelet testing and electron microscopy studies in adolescents with platelet function disorders

	Prolonged Closure Times^*^	Impaired Platelet Aggregation Responsiveness by Agonist^*^					EM Reduced DG/PL^*^
Patient Group	C-EPI, C-ADP	EPI	ADP	Ristocetin	AA	Collagen	<3.68 DG/PL
	(n=43)			(n=35^§^)			(n=28)
Heavy Menstrual Bleeding	18 (41.9)	14 (40.0)	10 (28.6)	8 (22.9)	5 (14.3)	2 (5.7)	26 (92.9)
	(n=20)			(n=20)			(n=6)
Non-Heavy Menstrual Bleeding	10 (50)	15 (75)	7 (35)	5 (25)	7 (35)	1 (5.0)	6 (100)

AA=arachidonic acid; C-EPI=collagen-epinephrine; C-ADP=collagen-ADP; DG/PL=dense granules per platelet; EM=electron microscope; ADP= adenosine diphosphate; epi=epinephrine.

*P*-values exceeded 0.05 for all differences between the 2 groups.

Data are presented as n (%).

Closure times were performed on platelet function analyzer system; Aggregation studies were performed by light transmission aggregometry; Dense granule.

enumeration was performed by whole mount EM of platelet samples.

*Prolonged closure times=2 S.D. above mean RI:C-EPI, 86-143s & C-ADP, 59-106s; Impaired aggregation=2 S.D. below mean RI for each agonist's concentration: ADP:

0.5μM (78.1-99.8%),10μM (82.8-100.3%); AA:0.5mmol/L (75.6-102.8%); Collagen: 2.0μg/mL(75.8-110.0%), Epi:60μM, (76.6-107.5%), 150μM (78.9-107.1%);

Ristocetin:0.5mg/mL (0.0-21.7%), 1.15mg/mL (75.9-107.1%),1.37mg/mL (80.1-104.9%). Normal dense granule numbers per platelet: 4–6.

^§^Based on available aggregation studies.

#### Platelet function analyzer

Prolonged closure times by the PFA system, collagen-epinephrine (C-EPI) >143s and/or collagen-adenosine diphosphate (C-ADP) >106s, were observed in 18 (41.9%) and 10 (50%) of HMB and non-HMB patients, respectively, with no significant differences identified between the 2 groups (*P*=.17). Patients were subsequently diagnosed with a PFD based on LTA and/or EM results.

#### Light transmission aggregometry

LTA studies were variable, with no definitive aggregation pattern emerging, even among adolescents sharing similar clinical and laboratory profiles. Overall, 21 of 35 (60.0%) tested HMB adolescents had reduced maximal platelet aggregation with ≥1 agonists. Of the 21, most (16; 45.7%) had multiple agonist-induced aggregation defects: 8 teens to 2 agonists and another 8 to 3 agonists, while 5 (14.3%) had a single agonist abnormality. In the non–HMB group, 15 (75%) had abnormal platelet aggregation to ≥1 agonists. Of these, 13 (65%) had multiple agonist defects: 7 patients to 2 agonists, 4 to 3 agonists, and 2 to 4. Only 2 (10%) had a single agonist defect. LTA was normal with all 5 agonists for 14 (40%) and 5 (25%) HMB and non-HMB patients, respectively.

The most common agonists to elicit abnormal platelet responsiveness in both patient groups were epinephrine (60 μM and/or 150 μM) followed by ADP (5 μM and/or 10 μM), while collagen (2 μg/mL and/or 5 μg/mL) was the least common. Additional agonist-induced aggregation defects occurred in the majority of HMB and non-HMB teens (64-93% epinephrine; 70-86% ADP), respectively, with only occasional isolated epinephrine (17-36%) and ADP (14-30%) defects.

Ristocetin (1.15 mg/mL and/or 1.37 mg/mL)-induced aggregation abnormalities uniformly occurred with additional agonist-induced defects in 8 (22.9%) and 5 (25%) HMB and non-HMB adolescents, respectively, and did not occur as an isolated impairment. The 0.5 mg/mL ristocetin did not adversely influence platelet aggregation in either group. Patients with the ristocetin defect were predominately Caucasian and VWF tests for VWD diagnosis were all normal [27]. Impaired arachidonic acid (AA) (0.5 mmol/L)-induced responsiveness was found in 5 (14.3%) HMB and 7 (35%) non-HMB adolescents, with all 12 diagnosed with aspirin-like-defect (ALD). The ALDs were not subtyped according to defects in the AA-pathway. However, 2 of the 12 adolescents with absent AA-induced responsiveness had impaired aggregation with other agonists and preserved ristocetin responsiveness, suggesting a thromboxane A2 receptor defect. Only 1 (HMB) adolescent had an isolated AA defect. There were no significant differences between the groups with respect to aggregation responsiveness for any of the 5 agonists (*P*>.05).

#### Electron microscopy

**Figure 1:**
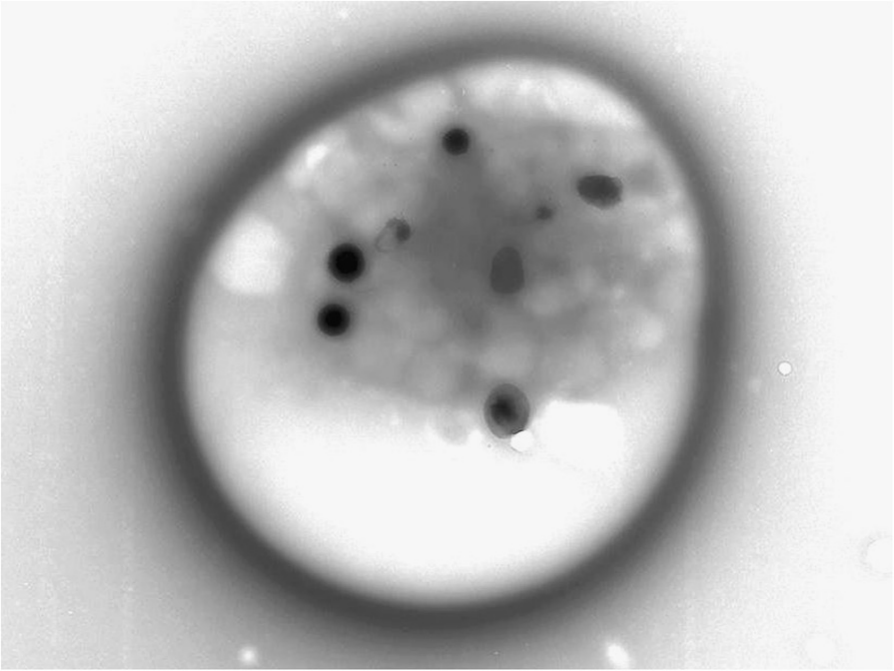
Transmission electron micrographic image of a normal platelet with 8 dense (δ) granules evident (whole mount EM, unstained; 10,000X).

EM detected significantly reduced platelet δ-granule numbers, <3.68DG/PL, in 26 (92.9%) of 28 tested HMB adolescents, while normal δ-granule numbers (4-6DG/PL) were calculated in 2 (7.1%) [Figure 1]. All 6 (100%) tested non-HMB adolescents had diminished platelet δ-granule numbers. EM identified one patient with the combined α-δ storage pool defect.

### Types of platelet defects

Platelet defect types are shown in Table 4 for both adolescent groups. Adolescents diagnosed with HMB had a significantly greater incidence, 32 (74%), of the δ-SPD than teens that did not manifest HMB (9; 45%) after menarche. Indeed, the proportion of the δ-SPD was 1.64-fold higher (RR 1.64, 95% CI 1.06, 2.92) and the prevalence odds ratio (OR) of the δ-SPD was 4.62-fold higher among HMB PFD teens than in their non-HMB counterparts (98% CI 1.47, 14.5, *P*=.007). The next most common PFD type was the ALD, diagnosed in 5 and 7 HMB and non-HMB PFD teens, respectively. One adolescent from each group was diagnosed with combined δ-SPD-ALD and one (HMB group) had the combined α-δ SPD. There were no cases of absent δ- or α-granules, as found in classic, syndromic platelet disorders, e.g., Hermansky-Pudlak Syndrome or gray platelet syndrome, respectively.

**Table 4:** Types of platelet defects identified in postmenarcheal adolescents

**Platelet Defect**	**Heavy Menstrual Bleeding (n=43)**	**Non-Heavy Menstrual Bleeding (n=20)**	***P*-value**	**Odds Ratio (95% CI)**
δ-SPD	32 (74)	9 (45)	0.007	4.62 (1.47,14.5)
Combined δ-SPD + Aspirin-like defect	1 ( 2)	1 ( 5)	>.05	
Combined α-δ-SPD	1 ( 2)	0	>.05	
Aspirin-like defect	4 ( 9)	6 (30)	>.05	
Platelet function disorder, NFC	4 ( 9)	4 (20)	>.05	
Combined PFD and type I, VWD	1 ( 2)	0	>.05	

α=alpha; δ-SPD=dense granule Storage Pool deficiency; NFC=not further classified; VWD=von Willebrand Disease.

Data are presented as n (%).

## Discussion

Platelet function disorders are a heterogeneous group of inherited bleeding defects with bleeding severity ranging from mild to severe. While some patients may be asymptomatic, most present with ecchymosis, epistaxis, HMB or excessive bleeding associated with surgical procedures or trauma. The present retrospective study includes the largest reported series on adolescents that clinically manifest HMB due to platelet function disorders (n=43). The study methodically evaluates in this population subgroup various clinical and laboratory characteristics not previously examined. Data from the study offer novel insights into the early natural history of the disorder that will assist in its characterization as well as earlier identification.

Observations from the present study intriguingly suggest that adolescents with PFDs that manifest HMB after menarche represent a clinically distinct phenotype from similarly affected teenagers that do not develop HMB after pubertal transition. Indeed the possibility that intra- and intergroup differences exist at the genomic or proteomic levels and these differences influence variable phenotypic bleeding manifestations is supported by the finding of a significantly higher incidence of the δ-SPD (74%) in adolescents with HMB when compared with non-HMB teenagers (*P*<.007).

The incidence of HMB was considerable among adolescents with documented PFDs. It was the most common bleeding symptom, manifesting in over two-thirds (68%) of young females affected with PFDs. Both the (mean) age-at-PFD diagnosis and the presenting symptomatology differed between the 2 groups. Young girls that developed HMB after menarche were significantly older when they were diagnosed with a PFD—by 3.6yrs—when compared with non-HMB teens (*P*<.01). Indeed, a significant majority (86%) were diagnosed with a PFD at or after the menarche based on HMB symptoms, while 14% were rendered a PFD diagnosis prior to the menarche based on associated bleeding, e.g., epistaxis, ecchymosis, etc. (*P*<.01) Overall, only 30% of patients diagnosed with a PFD prior to menarche subsequently manifested HMB after pubertal transition, and all had the δ-SPD, while a large majority (70%) did not develop HMB after menarche (42mo median follow-up).

Significant differences between age-at-PFD diagnoses may in part reflect bleeding severity. Individuals with severe bleeding symptoms often present early in childhood, suggesting patients that did not manifest HMB after menarche presented with more severe bleeding that led to earlier evaluation and diagnosis [28]. Some may have had family members with positive bleeding histories, prompting earlier evaluation. However, owing to the heterogeneity of PFDs, it is possible that some may not have manifested as severe bleeding symptoms as other patients with *de novo* PFD diagnoses, but without positive familial bleeding histories. Additionally, our HMB teens shared a similar high prevalence of family bleeding tendencies and yet were diagnosed with a PFD at an older age. Hemostatic challenges did not appear to influence earlier PFD diagnoses as evidenced by similar intergroup surgery-associated bleeding histories, particularly childhood tonsillectomies and adenoidectomies. Treatment regimens, such as desmopressin acetate, tranexamic acid, etc., may have prevented HMB. However, therapeutic intervention does not explain the existence in our study of a subset of young girls diagnosed with a PFD prior to menarche (14%) that developed HMB after transitioning into puberty. Indeed, similar findings were recently reported in a study by Chi et al. in which the diagnosis of a bleeding disorder was known in 64% of young females prior to their presentation with menorrhagia, yet they developed HMB despite instruction to take tranexamic acid when menses began [14].

The long (mean) 1.8-year interval between menarche-to-HMB onset was at variance with prior studies on menorrhagia that reported HMB occurring predominately at or within the first year of menarche in patients with bleeding disorders [4,6,7,9]. Indeed, Chi et al. reported HMB onset occurred at menarche in 90% of adolescents affected with bleeding disorders [14]. Adolescents with PFDs that clinically manifested HMB have not been exclusively analyzed until the present report. It is possible that differences in the menarche-to-HMB interval may be related in part to prior studies intermixing data from PFD patients with data from subjects affected with disparate bleeding disorders, thus obscuring the actual temporal relationship [4,5,7,14]. Of interest, PFD adolescents that clinically manifested HMB were not anemic. For these patients, the menarche was a recent event with the PFD diagnosis rendered close to the menstruation onset. This may have aided in earlier treatment initiation that may have in turn mitigated heavy bleeding and subsequent anemia.

A substantial number of adolescents with PFDs, 70-76%, had blood type O, intriguingly paralleling the 77% type O frequency reported for VWD, type I individuals [29,30]. Indeed, the high blood type O phenotypic frequency was an unexpected finding and to our knowledge has not been previously reported. When examined in the context of a comparable U.S. population, blood group O occurred in our PFD adolescents significantly more frequently and types A and B, less frequently (*P*<.037) [21,22]. Prior studies on menorrhagia have reported type O blood occurring in 44-59% of their patients, paralleling national norm frequencies, despite their inclusion of numerous VWD females [8,9,11,12]. Blood type O individuals have VWF levels approximately 25% lower than non-type O individuals. Our patients had normal ABO-adjusted VWF testing with only one patient, with combined PFD-VWD, type I showing VWF studies diagnostic for VWD diagnosis. The mechanism for the preponderance of blood group O, in the absence of VWD in our PFD adolescents is unknown [29,30]. Indeed, mechanisms influencing blood group O on plasma VWF levels are elusive. Variable VWF carbohydrate structures have been associated with lowered VWF levels in blood group O individuals. Additionally, blood group O individuals have significantly higher rates of VWF proteolysis by the metalloproteinase, a disintegrin and metalloproteinase with thrombospondin motif, member 13 (ADAMTS13) when compared with non-O individuals [31]. Genetic differences in ADAMTS13 levels may also influence VWF clearance in type O blood groups. Lower VWF levels (not diagnostic for VWD) as reported in blood type O individuals, combined with functional platelet defects, particularly the δ-SPD, may represent an additional variable adversely affecting hemostasis that renders PFD-blood type O individuals more vulnerable to bleeding than PFD patients expressing A, B blood types.

The significant association between adolescents with HMB and PFD, particularly the δ-SPD, suggests that blood type O may be a useful identifier for these disorders and may assist clinicians in stratifying patients for additional studies. Certainly, blood typing is not intended to serve as a screening test for bleeding disorders. However, if blood typing has been performed, data from our study indicate adolescents presenting with HMB that express blood type O, have normal coagulation profiles and ABO-adjusted VWF tests for VWD, and platelet aggregation studies that are either normal or impaired by a single agonist defect may benefit from additional EM studies to exclude the δ-SPD. Without a definitive diagnosis, such as that offered by EM, many patients with this disorder may go undetected.

Prior reports and data from our study indicate that standard platelet screening by closure times (PFA system) and LTA have moderate sensitivity in detecting and diagnosing PFDs, particularly the δ-SPD [15,26,32-36]. The Hayward et al. 2006 report of a survey on published literature regarding PFA efficacy concluded that the test lacked adequate sensitivity in screening for platelet disorders [32]. Philipps and coworkers studied the utility of closure times (PFA) and bleeding times in screening for bleeding disorders in women with HMB. Their data indicated the PFA had a sensitivity of 23%, specificity of 92%, positive predictive value of 75% and a negative predictive value of 52% in women with PFDs [35]. Cattaneo et al. studied closure time (PFA system) and bleeding time effectiveness in patients with δ-SPDs and platelet secretion defects and concluded that both tests performed with similarly low sensitivity [36]. Data from these reports supports our finding of high false negative closure times.

Platelet LTA, considered the “gold standard” for diagnosing PFDs, has well-known limitations [26,37]. The issue of whether single agonist-induced aggregation defects can accurately detect a PFD is a potential limitation. Most patients with PFDs, 29.7% - 30.3%, in 2 prior studies on HMB had single agonist defects identified by platelet aggregation studies. In our study, the majority (45.7%) of HMB patients had multiple agonist-induced aggregation defects, while only 14.3% had a single agonist abnormality and our non-HMB group showed similar testing results. Hayward et al. reported that reduced maximal aggregation with >1 agonists significantly increases PFD detection [12,15,38]. Miller et al. recently addressed the criterion of >1 agonist defects and the subject of multiple statistical comparisons used in platelet function testing. The authors noted that had the >1 agonist criterion been applied in their study, 30.3% of patients would have been excluded, as would those patients affected with a single platelet receptor defect to one agonist (e.g., collagen, ADP) [15]. In our study, 14.3% of HMB patients with single agonist defects would have been similarly excluded, all of whom were diagnosed with the δ-SPD by EM. Indeed, these 5 patients with the following LTA single agonist defects: 0.5 μM ADP (3); 60 μM epinephrine (1); and 0.5 mmol/L AA (1) all had significantly reduced platelet δ-granules on EM. Miller et al. appropriately cautioned against rendering a PFD diagnosis on the basis of a single test [15]. However, some patients with the δ-SPD have single agonist aggregation defects, as identified in 14.3% in our study, as well as normal aggregation studies. Nieuwenhuis et al. reported on 106 patients with platelet disorders with 25% having normal platelet aggregation studies and all were subsequently diagnosed with the “SPD” by total platelet ADP and serotonin testing [33]. Israels et al. similarly reported on a subset of 15 patients with normal aggregation studies subsequently diagnosed with “platelet storage pool deficiency” by EM and ATP release testing [34]. These studies parallel our findings. Indeed, the δ-SPD diagnosis would have been missed in an additional 25.6% and 10% of our HMB and non-HMB teenagers, respectively, that had normal standard platelet function studies (closure times by PFA; LTA) and for whom EM studies revealed significantly reduced platelet δ-granule numbers.

EM may be indicated in some cases as a confirmatory method for detecting diminished granule numbers [23-26,34]. Although not widely available, other diagnostic studies for the δ-SPD, such as flow cytometry of mepacrine uptake, are similarly not widely available. Clinical judgment should be rendered; as previously stated, data from our study indicate that young girls with HMB, normal coagulation profiles and ABO-adjusted VWF tests for VWD, blood type O, and normal or single agonist defects on platelet function studies, would be excellent candidates for EM studies for detecting the δ-SPD. EM studies are done at a number of centers. Excellent distinctions between normal and diminished δ-granule numbers have been reported among those institutions included in a recent study by Hayward et al. [26].

Limitations to the study include those common to retrospective reports. There was an absence of an adolescent control group with unexplained HMB after exclusion of all non-hematologic etiologies. We sought in part to define the clinical profile of PFD adolescents at presentation and no attempts were made to correct for PBAC changes over time and after treatment. Sanitary products were not standardized and this is an infrequent limitation [4-8,11-15]. A small minority of HMB patients did not have platelet LTA results recorded, with only observed data used in the analyses and this is an infrequent occurrence [4,5]. The potential for selection bias in our specialist-referred patient population and cases sent for EM cannot be excluded. A potential confounder is whether timing of platelet samples sent for EM either during or shortly after a hemostatic challenge affected δ-granules numbers. Exhausted platelet granules would be expected to adversely influence platelet aggregation studies, yet 40% of adolescents with reduced δ-granule numbers on EM had normal platelet aggregation studies. Despite these limitations, detailed clinical and laboratory data obtained from the study has assisted in defining the characteristics of adolescents with platelet dysfunction-associated HMB that have never before been reported on. Data from the study provides a foundation for future longitudinal cohort studies. Additionally, these data will aid clinicians in earlier diagnosis of the disorder and will alert them to select additional platelet testing, such as EM, particularly in the presence of normal standard platelet function studies.

## Methods

### Patients

Retrospective review of medical records of young females with documented PFDs referred to the West Central Ohio Hemophilia Treatment Center and pediatric-adolescent gynecology faculty practices at Dayton Children’s Medical Center and the Miami Valley Hospital for evaluation and routine follow-up for bleeding diatheses between June 1998 and June 2009. Institutional Review Boards at the centers approved the study and patient confidentiality was protected according to HIPAA guidelines.

Collected demographic information included: age at PFD diagnosis; age at presentation; age at menarche; age(s) at onset of HMB and/or other bleeding symptoms; ethnicity; medical and surgical histories; family history of bleeding tendencies; medication history; physical examination; laboratory studies; consultations; and radiographic and/or other diagnostic studies. Records were also reviewed for a physician’s diagnosis and clinical history of HMB along with supporting documentation, including assessment of menstrual blood loss using the PBAC. Physical examination findings and laboratory studies were scrutinized for any tangible manifestations or biochemical changes associated with anovulation and/or polycystic ovary syndrome including, but not limited to, hirsutism, acne and/or obesity as well as clinical phenotypes associated with syndromic platelet disorders. Patients with any laboratory studies suggestive of an existing endocrine disorder or any other known medical condition, iatrogenic anticoagulation, medications interfering with platelet function or anatomical defects that resulted in HMB were excluded. Records from postmenarcheal patients with documented PFDs were set aside for further consideration.

The study population was composed of a cohort of postmenarcheal adolescents evaluated for bleeding diatheses and rendered a diagnosis of PFD between ages (range) 3.0-20.17 years. Patients were followed at 6-month intervals for up to 11 years with 42 months the median duration. Patients were evaluated and stratified into 2 groups based on the presence or absence of a physician’s diagnosis of HMB and supporting documentation. Patient stratification allowed in patients affected with the similar disease process of platelet dysfunction investigation of both disparate and common characteristics that would assist in distinguishing the disorder of platelet dysfunction-associated HMB.

At menarche, all patients were interviewed regarding the menstrual cycle and were provided with standard institutional menstrual booklets that included PBACs. The booklets were returned and reviewed by one hemophilia center nurse coordinator (ND) at each visit as previously described [39]. Briefly, patients were not provided with pads or tampons, but were instructed on proper methods for recording the menses duration and interval; estimation of blood loss and clot sizes; the number of disposed pads and/or tampons used per menses; and PBAC completion. The PBAC semi-quantitative scoring system was used to approximate menstrual blood loss, with a score ≥100 corresponding to >80 mL of menstrual blood loss per cycle and correlates with the definition of HMB [22,23]. While spectrophotometric analysis of alkaline hematin at 450 nm is considered the gold standard for quantifying menstrual blood loss, the PBAC is a reasonable, uncomplicated and validated method that is often used [40,41]. Patient histories of HMB were recorded; documentation consisted of a recorded mean PBAC score ≥100. Mean PBAC scores <100 were not in accordance with a HMB diagnosis. Two attending physicians (JAF, LSA) or a hemophilia center nurse coordinator (ND) reviewed all PBAC scores.

### Laboratory studies

All blood samples were obtained using standard phlebotomy techniques. Hemostatic testing was performed by one specialized coagulation laboratory. Hematologic studies for all patients were conducted as previously published [21]. Briefly, initial testing consisted of: CBC with platelet count and examination of platelet morphology, PT, aPTT, ABO-Rh(D) blood group typing, fibrinogen, VWF:RCo, VWF:Ag, VWF:RCo / VWF:Ag ratios and FVIII:C. Selected patients underwent single coagulation factor II, V, VII, IX, XI and/or XII activity levels. Patients with normal platelet counts and morphology, ABO-adjusted ranges for VWF levels, coagulation studies, fibrinogen levels and single coagulation factor activity levels underwent platelet function studies and/or subsequent electron microscopy (EM) quantification of platelet dense (δ) granules or qualitative investigation of alpha (α) granules.

### Platelet function studies

Patients were instructed to avoid prostaglandin inhibitors and all other medications interfering with platelet function for 10-14d prior to platelet studies.

#### Platelet function analyzer closure times

Whole blood samples were collected in 3.2% citrate and processed within 2hrs of collection. Closure times (CTs) using the platelet function analyzer system (PFA-100^®^, Dade Behring, Inc., Miami, Florida) were conducted on patient samples with C-EPI and C-ADP coated cartridges with respective reference intervals (RIs), 86-143sec and 59-106sec. RIs were obtained from normal females that abstained from platelet function-interfering medications 10-14d prior to testing and were determined as 2 standard deviations (S.D.) about the mean. Normal controls were run with each new cartridge lot and testing was performed in duplicate. Closure times were considered prolonged, with either one (C-EPI or C-ADP) or both cartridges, when the results were more than 2 S.D. above the mean and repeat testing confirmed the abnormality.

#### Light Transmission Aggregometry

Light transmission aggregometry was performed on platelet-rich plasma (PRP) using the PACKS-4 aggregometer (Helena Laboratories Corp., Beaumont, Texas) and specimens were processed within 3hrs of collection. Activation of PRP was performed in accordance with the manufacturer’s guidelines and spontaneous platelet aggregation was observed. RIs were calculated as 2 S.D. about the mean in normal female controls. The following agonists were added at the stated final concentrations included with their respective RIs for maximal percentage of platelet aggregation: adenosine diphosphate (ADP), 0.5 μM (78.1-99.8%) and 10 μM (82.8-100.3%); arachidonic acid (AA), 0.5 mmol/L (75.6-102.8%); collagen 2.0 μg/mL (75.8-110.0%) and 5.0 μg/mL (83.3-103.2%); epinephrine, 60 μM (76.6-107.5%) and 150μM (78.9-107.1%); and ristocetin, 0.5 mg/mL (0.0-21.7%,), 1.15 mg/mL (75.9-107.1%) and 1.37 mg/mL (80.1-104.9%). Maximal percentage for platelet aggregation responsiveness was measured over 10min duration. Platelet LTA studies were considered impaired when maximal aggregation responsiveness was reduced more than 2 S.D. below the lower RI with ≥1 agonists. Test results were reported as either in range or out of range, with all LTA tracing examined by one hematologist (JAF).

Patients having normal results from platelet function studies (closure times by PFA; LTA) or a single agonist-induced aggregation defect on LTA together with a strong clinical suspicion of an underlying bleeding disorder and/or a family history positive for bleeding tendencies were selected to undergo platelet EM studies.

#### Electron microscopy

EM studies on patient platelet samples were assessed for quantitatively reduced δ-granules and qualitative α-granule reduction. Whole mount EM morphologic examination was used to quantify of platelet δ-granules. Collected samples were prepared by placing one drop of citrated-PRP on parlodian-coated grids for 5min followed by 2 distilled water rinses. Filter paper was used to blot excess fluid from the grid edge. After air-drying at room temperature, platelets were examined by one expert (WTG) using a FEI Tecnai transmission EM (FEI, Hillsboro, Oregon) without fixation or staining.

Classification of δ- and α-granules was based on morphological parameters elucidated by Weiss et al. and White et al. [23,24]. Platelet α-granules were investigated qualitatively. Enumeration of δ-granules was performed as previously described [24,25]. The mean number of δ-granules identified in 100 contiguous platelets was reported. Dense granule (DG) diameters were determined and DG mean volume (v = 4/3r^3^) was calculated from at least 10 platelets (30–100 DGs analyzed). The total volume of dense granules (TDGV) per platelet was determined by multiplying the mean dense granule volume (DGV) × dense granule number (DGN) [25]. Significantly reduced mean δ-granules/platelet (DG/PL) numbers and/or total δ-granule volume/platelet (TDGV/PL) were calculated as <3.68 DG/PL or <8.0 × 106fL, respectively, from that determined in normal female controls (4–6 DG/PL or 8–12 × 106fL) [23-25].

Diagnostic criteria for a PFD included reduced maximal platelet aggregation responsiveness with ≥1 agonists 2 S.D. below RI on LTA in the absence of platelet function-interfering medications and/or EM detection of reduced δ-granules numbers, or near absent α-granules with replacement vacuolization. The δ-SPD was rendered in some cases only by EM studies [11,12,23-26]. Diagnostic criteria for the aspirin-like defect (ALD*)* included absent to significantly reduced (≤10%) AA-induced platelet responsiveness, irrespective of additional epinephrine and/or ADP-induced aggregation abnormalities.

### Statistical Analysis

All data from postmenarcheal adolescents with documented PFDs, one group diagnosed with HMB and the other without a HMB diagnosis, were analyzed using SAS statistical package, version 9.2 (SAS Institute, Inc., Cary, North Carolina). The means and S.D. for ages at PFD diagnosis, menarche, and HMB; intervals from age-at-PFD diagnosis to age-at-menarche and age-at-menarche-to-age-at-HMB onset; and laboratory testing parameters were calculated. RIs for platelet testing were calculated as 2 S.D.s about the mean for normal female controls as previously stated. For continuous variables, a t-test was used to compare the means between the two groups. The *Χ*^2^ test was used for categorical variables to determine whether an association existed between the two groups. A finding was considered statistically significant if *P* was ≤.05. Odds ratios (OR) and 95% confidence intervals (CIs) were used to compare the δ-SPD prevalence between both groups.

## Abbreviations

AA: Arachidonic acid; ADAMTS13: a disintegrin and metalloproteinase with thrombospondin motif, member 13; ADP: Adenosine diphosphate; ALD: Aspirin-like defect; aPTT: Activated partial thromboplastin time; C-ADP: Collagen-adenosine diphosphate; CBC: Complete blood count; C-EPI: Collagen-epinephrine; CI: Confidence interval; CT: Closure time; δ: Dense granule; δ-SPD: Dense granule storage pool deficiency; DG/PL: Dense granules per platelet; DGN: Dense granule number; DGV: Dense granule volume; EM: Electron microscopy; F VIII: Factor VIII activity; HMB: Heavy menstrual bleeding; LTA: Light transmission aggregometry; MCV: Mean corpuscular volume; PBAC: Pictorial blood assessment chart; PFD: Platelet function disorder; PFA: Platelet function analyzer; PL: Platelet; PTT: Prothrombin time; RI: Reference interval; S.D.: Standard Deviation; SPD: Storage pool deficiency; TDGV: Total volume of dense granules; VWD: von Willebrand disease; VWF: von Willebrand factor; VWF:RCo: Ristocetin cofactor; VWF:Ag: VWF antigen.

## Competing interests

The authors declare that they do not have competing interests.

## Authors’ contributions

Conception and study design, data acquisition: all authors; data analysis and interpretation: LSA, TPA, WTG, JAF; drafting the manuscript and revising it critically for important intellectual content: LSA, TPA, WTG, ND. All authors read and approved the final manuscript.
